# The Use of a Novel Endotracheal Tube and Airway Management System to Prevent Complications in Lung Transplantation

**DOI:** 10.7759/cureus.5170

**Published:** 2019-07-18

**Authors:** Vivian Hernandez-Torres, Robert Ratzlaff, Mathew Thomas, Tathagat Narula, Archer K Martin

**Affiliations:** 1 Department of Anesthesiology and Perioperative Medicine, Mayo Clinic, Jacksonville, USA; 2 Department of Critical Care, Mayo Clinic, Jacksonville, USA; 3 Department of Cardiothoracic Surgery, Mayo Clinic, Jacksonville, USA; 4 Department of Transplantation, Mayo Clinic, Jacksonville, USA; 5 Department of Cardiovascular and Thoracic Anesthesiology, Mayo Clinic, Jacksonville, USA

**Keywords:** lung transplantation, transplantation complications, perioperative management, mechanical ventilation

## Abstract

A key component of the perioperative management of lung transplant recipients is the avoidance of airway and pulmonary complications in the immediate postoperative period. The AnapnoGuard™ AG100s (Hospitech Respiration Ltd, Kfar Saba, Israel), a novel endotracheal tube and ventilation management system, holds the potential to assist the care team in attenuating complications related to excessive cuff pressure, subglottic secretions, and endobronchial intubation. In this report, we describe the successful use of the AnapnoGuard™ AG100s system in the postoperative management of a lung transplant recipient.

## Introduction

Lung transplantation is the definitive treatment for patients with end-stage pulmonary disease [1]. However, major risks and postoperative complications can occur following transplantation, some of them being serious and resulting in a high mortality rate [2]. Pulmonary complications may occur during the immediate postoperative period and include airway complications such as anastomotic and non-anastomotic stenosis, dehiscence, torsion, and pneumothorax [3]. In addition, a postoperative course including prolonged mechanical ventilation increases the risk of bacterial colonization and infections [4].

The AnapnoGuard™ AG100s system (Hospitech Respiration Ltd, Kfar Saba, Israel) is a novel endotracheal tube (ETT) and airway management device for use in intubated patients with a promising role in the prevention of both airway and pulmonary complications after lung transplantation. The system provides continuous monitoring of leaks around the cuff based on the carbon dioxide (CO_2_) level in the subglottic space. At the same time, the system automatically performs programmable subglottic suction of secretions via synchronized, simultaneous rinsing with saline and suction [5]. The authors present a lung transplantation case that was placed on the AnapnoGuard™ AG100s system in the postoperative period with no complications.

## Case presentation

A 53-year-old patient presented for bilateral orthotopic lung transplantation due to idiopathic pulmonary fibrosis. Initial intraoperative endotracheal intubation was achieved with the use of a left-sided double-lumen tube (DLT), and the case proceeded with central veno-arterial extracorporeal membrane oxygenation (VA ECMO) support. The patient was weaned off VA ECMO, with subsequent decannulation and chest closure. Before leaving the operating room, the DLT was exchanged to a single lumen AnapnoGuard™ ETT, and the patient was transferred to the intensive care unit on a transport ventilator. Immediately upon arrival, the patient was connected to the AnapnoGuard™ AG100s system for protective airway management. The patient was connected for four days, 15 hours, and 56 minutes without complications and the system was operated the whole period with constant regulation of ETT cuff pressures. During this time, ETT cuff pressures limits were held between 26 and 28 cm H_2_O except during secretion removal. A total of 50 cc of secretions were removed for the duration of intubation, and the leak test was performed 11 times, with no CO_2_ leaks detected. Of note, the postoperative care team was able to perform routine post-transplantation bronchoscopies via the ETT without event. On post-surgical day five, the patient failed trials with T-piece due to right diaphragmatic paralysis and underwent a tracheostomy. Eventually, diaphragmatic function recovered and the patient was successfully decannulated.

## Discussion

**Figure 1:**
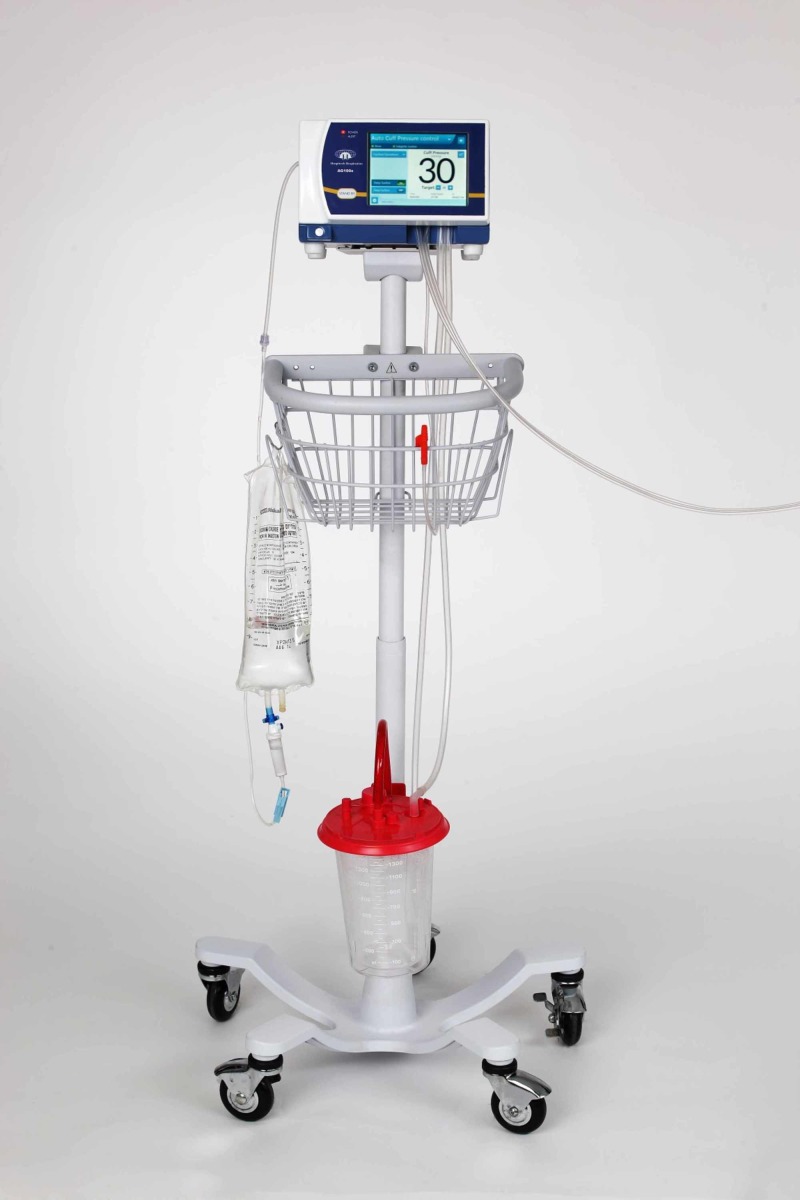
AnapnoGuard™ AG100s System. Used with permission from Hospitech Respiration Ltd. AnapnoGuard™ AG100s system (Hospitech Respiration Ltd, Kfar Saba, Israel)

**Figure 2:**
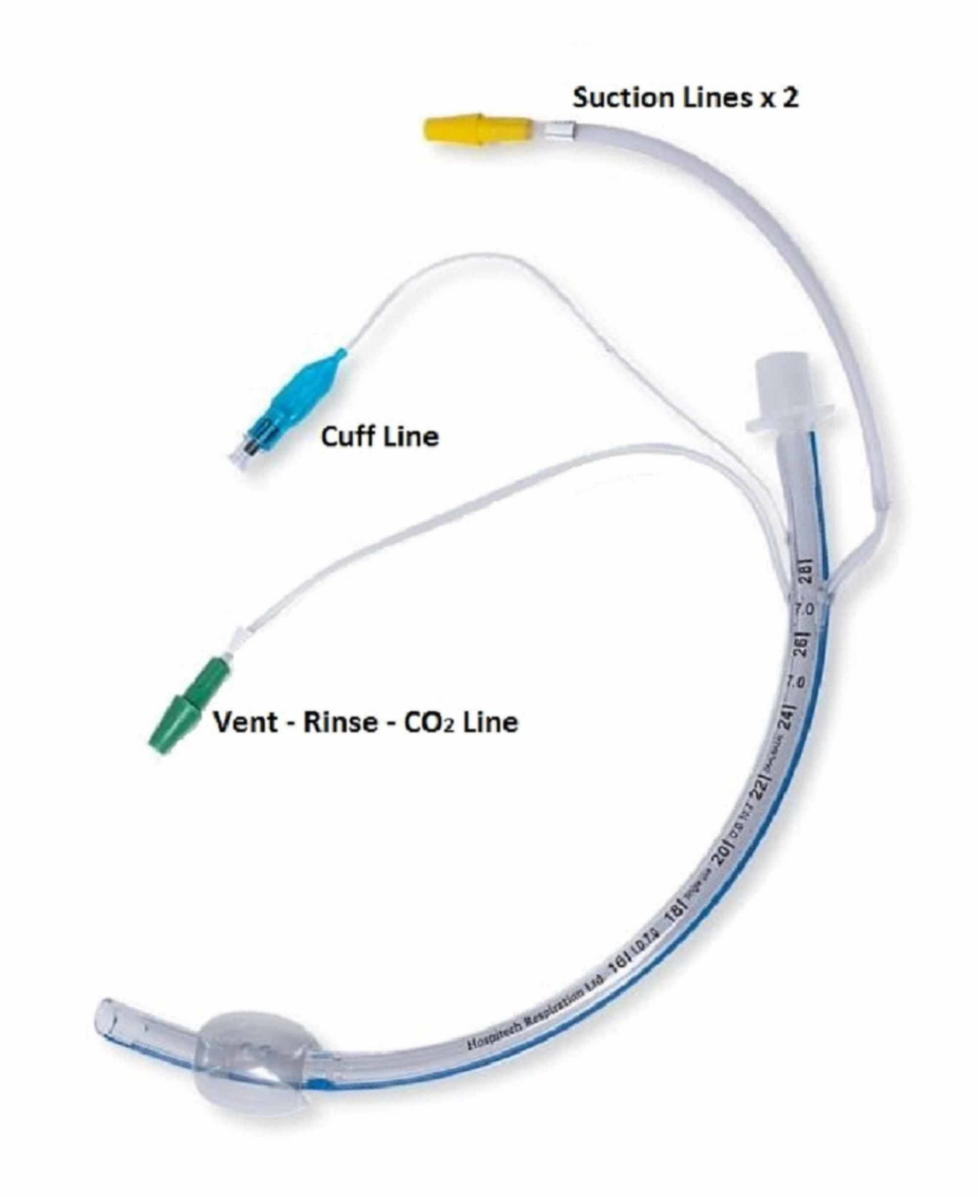
AnapnoGuard™ Endotracheal Tube. Used with permission from Hospitech Respiration Ltd. AnapnoGuard™ (Hospitech Respiration Ltd, Kfar Saba, Israel)

The AnapnoGuard™ AG100s system consists of a single lumen ETT (see Figure 1), a connection kit, and a control unit (see Figure 2). The ETT is a polyvinylchloride ellipsoidal tube with a thin wall polyurethane cuff, two suction lines, and an extra CO_2_ venting line [5]. The ETT is connected to the AG100s control unit, which provides continuous cuff pressure regulation, irrigation, and suctioning via programmable cycles [5]. The device operates automatically within these cycles, where air samples from the subglottic space are taken to measure CO_2_ levels and determine whether or not the cuff is sealing the trachea. The system keeps the target pressure constant, adjusting cuff pressure based on the presence or absence of CO_2_ levels above the cuff. If a leak is detected, the system responds by inflating the cuff to the target pressure. If a CO_2_ leak is not detected, the cuff pressure is reduced by 1 cm H_2_O and is maintained using an automatic feedback loop to ensure effective sealing at minimal ETT cuff pressure [5]. Additionally, the system automatically performs programmable evacuation of airway secretions from the subglottic space via a combination of dual suction lumens and irrigation of saline to facilitate secretion removal [5]. This system has received Food and Drug Administration (FDA) and Conformité Européene (CE) approval and has been reported as a method of potentially attenuating complications related to mechanical ventilation [5-7].

The application of the AnapnoGuard™ AG100s system in the lung transplantation population has the potential to attenuate airway and pulmonary complications in the immediate postoperative period by the constant regulation of ETT cuff pressure, removal of secretions, and ability to detect endobronchial intubation. ETT cuff pressure should be regulated to provide an adequate seal while avoiding excessive pressure in order to reduce the risk of complications [8]. A minimal pressure of 20 cm H_2_O is recommended to prevent aspiration and ventilator-associated pneumonia while a maximal of 30 cm H_2_O has been reported to be associated with impaired tracheal wall blood supply [9]. Other complications associated with excessive pressure include postoperative hoarseness, subglottic stenosis, nerve damage, fistulas, or tracheal wall damage [9-12]. Additional safety components of the system involve suctioning airway secretions and detection of endobronchial intubation, which are key for transplanted lung graft protection by helping to prevent ETT-associated infections and diminish the risk of injury to anastomotic suture lines [13].

## Conclusions

The AnapnoGuard™ AG100s system is an airway management device that provides continuous cuff pressure monitoring and subglottic secretion removal in mechanically ventilated patients. When using this device in our patient, we observed quantitative removal of secretions, lack of cuff leak, and successful use of postoperative surveillance bronchoscopies via the AnapnoGuard™ ETT. Future clinical studies are needed to determine the impact of this system on airway and pulmonary complications after lung transplantation.
